# A-Methylacyl-CoA Racemase (AMACR) and Prostate-Cancer Risk: A Meta-Analysis of 4,385 Participants

**DOI:** 10.1371/journal.pone.0074386

**Published:** 2013-10-09

**Authors:** Ning Jiang, Shimiao Zhu, Jing Chen, Yuanjie Niu, Liqun Zhou

**Affiliations:** 1 Department of Urology, 2nd Hospiital of Tianjin Medical University, Tianjin Institute of Urology, Tianjin, China; 2 Department of Urology, Peking University First Hospital, The Institute of Urology, Peking University, Beijing, China; University of Kentucky College of Medicine, United States of America

## Abstract

**Background:**

Alpha-methylacyl-CoA racemase (AMACR) is a mitochondrial and peroxisomal enzyme that is overexpressed in prostate cancer. The aim of this study was to confirm and expand the findings that the PCa risk increased in men associated with AMACR expression across various geographic regions.

**Methods:**

A systematic search of databases was carried out and other relevant articles were also identified. Then the meta-analyses were conducted according to the standard guidelines.

**Results:**

A total of 22 studies with 4,385 participants were included on the basis of inclusion criteria. AMACR by IHC was significantly associated with increased diagnosis of PCa (OR = 76.08; 95% CI, 25.53–226.68; *P*<0.00001). Subgroup-analysis showed that findings didn’t substantially change when only Caucasians or Asians (OR = 51.23; 95% CI, 19.41–135.24; *P*<0.00001) were considered. Expression of AMACR by PCR in relation to PCa risk suggested that AMACR was associated with PCa (OR = 33.60; 95% CI, 4.67–241.77; *P*<0.00001). There was also no significant publication bias observed.

**Conclusions:**

Our findings provide further evidences that the expression of AMACR contribute to PCa risk. AMACR protein overexpression was found in prostate cancers, low expression in any of the normal tissues or in benign prostatic tissue. AMACR is potentially an important prostate tumor marker.

## Introduction

Prostate cancer (PCa) is the most frequently diagnosed non-cutaneous malignancy in men, and the second leading cause of male cancer-related mortality in the United States [1]. The incidence of prostate cancer in Asia, including in China and Japan, has been increasing, although it is lower than that in the Western world [2]. Diagnosis of prostate cancer glands can sometimes present a diagnostic challenge for pathologists, since prostate carcinoma can mimic benign prostate glands [3]. and the architectural or cytologic clues for the diagnosis of carcinoma may not always be seen in small foci of suspicious glands. Also, Tissue diagnosis of prostate cancer can be difficult in needle biopsies or in a small focus of cancer of radical prostatectomies, presenting one of the major challenges in surgical pathology. underdiagnosis of a small focus of prostatic adenocarcinoma might delay early treatment and cause severe adverse consequences for patients. Therefore, a PCa specific marker could be be of great importance and usefulness to adjunct to facilitate critical diagnostic decisions with high sensitivity and specificity [4].

Although prostate-specific antigen (PSA) is the main criteria for PCa diagnosis, it has poor specificity to cancer, highly expressed in noncancerous prostatic tissues as well as in cancerous tissues and often lead to over diagnosis and overtreatment. Consequently a new scenario is needed to identify potentially aggressive or lethal PCa to better support clinical decisions [5].

AMACR (alpha-methylacyl-CoAracemase), an enzyme currently used in prostate cancer diagnosis, which is a peroxisomal and mitochon drial enzyme that was preferentially overexpressed to approximately 80% of prostate cancer detected in prostate biopsies [6]–[7]. However, AMACR is not 100% sensitive, and its expression is not limited to prostatic adenocarcinoma but may also be seen in several of its histologic mimics [8], resulting in many potential caveats in its use [9]. Accordingly, evaluation of AMACR as new markers of prostatic adenocarcinoma is needed.

In an attempt to confirm the potential role of AMACR expression as a prognostic biomarker, we completed a meta-analysis of AMACR expression in men of Asia and European lineage across different geographic regions with PCa.

## Evidence Acquisition

### Search Strategy and Selection Criteria

We undertook a comprehensive literature review with search terms (Table 1) without language restriction. We restricted the search to Medline, Web of Science and the Cochrane Library. The last quest was updated on March 13, 2013. Bibliographies of relevant retrieved studies and recent reviews were also scanned for additional publications. When more than one studies with the same population were identified, only the most recent or complete one was included in this meta-analysis.

**Table 1:** Characteristics of trials included in meta-analyses.

Study	Year	methods	Ethnicity	Cases		Controls		Study design	Control source
				Positive Total		Positive Total			
Rogers [14]	2004	IHC	Caucasian	12	17	0	7	cohort	biopsy negative
Shah [15]	2013	IHC	Caucasian	48	51	2.5	3	cohort	benign control
Trpkov [16]	2009	IHC	Caucasian	120	124	16	20	cohort	biopsy negative
Zhou [17]	2004	IHC	Caucasian	176	215	4	11	case series	benign control
Kaic [18]	2009	IHC	Caucasian	9	16	0	4	case series	benign control
Farinola [19]	2004	IHC	Caucasian	16	23	2	16	cohort	benign control
Puebla-Mora[20]	2006	IHC	Caucasian	37	41	6	22	cohort	benign control
Pertega-Gomes [21]	2013	IHC	Caucasian	270	349	12	203	cohort	benign control
Browne [22]	2004	IHC	Caucasian	40	44	2	33	cohort	benign control
Nassar [23]	2005	IHC	Caucasian	34	38	0	15	case series	benign control
Jiang [24]	2005	IHC	Caucasian	78	82	0	56	case series	benign control
Stewart [25]	2007	IHC	Caucasian	272	320	0	292	case series	benign control
Yamada [26]	2013	IHC	Asia	42	60	9	19	cohort	biopsy negative
Chen G [27]	2004	IHC	Asia	71	78	3	68	case series	benign control
Xiao [28]	2004	IHC	Asia	103	105	19	135	case series	benign control
Ng [29]	2007	IHC	Asia	111	113	4	134	case series	benign control
Yu [30]	2007	IHC	Asia	42	42	0	30	case series	benign control
Zielie [31]	2004	RT-PCR	Caucasian	7	10	9	9	case series	benign control
Jiang Z [32]	2004	RT-PCR	Caucasian	441	454	254	277	case series	benign control
Kristiansen [33]	2008	RT-PCR	Caucasian	583	614	0	31	case series	benign control
Schostak [34]	2006	RT-PCR	Caucasian	37	57	8	55	case series	benign control
Ouyang [35]	2008	RT-PCR	Caucasian	30	43	14	49	case series	benign control

IHC = Immunohistochemistry; RT-PCR = Reverse Transcription-Polymerase Chain Reaction.

Studies were included if they fulfilled the following criteria: 1) cases were pathologically verified to have adenocarcinoma of the prostate (International Classification of Diseases-10: C61), 2) the control group consisted of subjects who were men and free of PCa, 3) studies investigating the association of AMACR with PCa risk as the main outcome.

### Data Extraction and Quality Assessment

This meta-analysis was conducted according to the Preferred Reporting Items for Systematic Reviews and Meta-Analyses (PRISMA) [10] and Meta-analysis of Observational Studies in Epidemiology (MOOSE) [11] guidelines.

Study characteristics, ethnicity of included subjects, numbers of cases and control subjects, and positive staining were extracted for factors of interest. The authors of published studies were also contacted for requesting necessary data that were not provided. Quality assessment was undertaken independently by at least two authors (Ning Jiang, Shimiao Zhu, Jing Chen). Two authors (Liqun Zhou, Yuanjie Niu) independently did the literature search and extracted data. Any disagreements were resolved through discussion with authors (Niu and Zhou).

### Data Analysis and Presentation

The effect estimates of choice were odds radio (OR) for dichotomous variables and the corresponding 95% confidence intervals (CI). The random effects model of DerSimonian and Laird was prespecified for use in all estimates because of the suspected a priori that studies were conducted by various authors with different populations and had different designs (eg, case-control and case series studies). Heterogeneity was evaluated using the Q test [12]. We also calculated the quantity *I* ^2^ statistic that represented the percentage of total variation across studies. As a guide, *I* ^2^ values of 25%, 50%, and 75% correspond to low, medium, and high levels of heterogeneity [13]. The funnel plot was addressed to reveal the potential publication bias. All analyses were conducted using Review Manage, version 5.2 (The Cochrane Collaboration, Oxford, U.K.).

## Evidence Synthesis

### Literature Search and Characteristics of Studies

**Figure 1:**
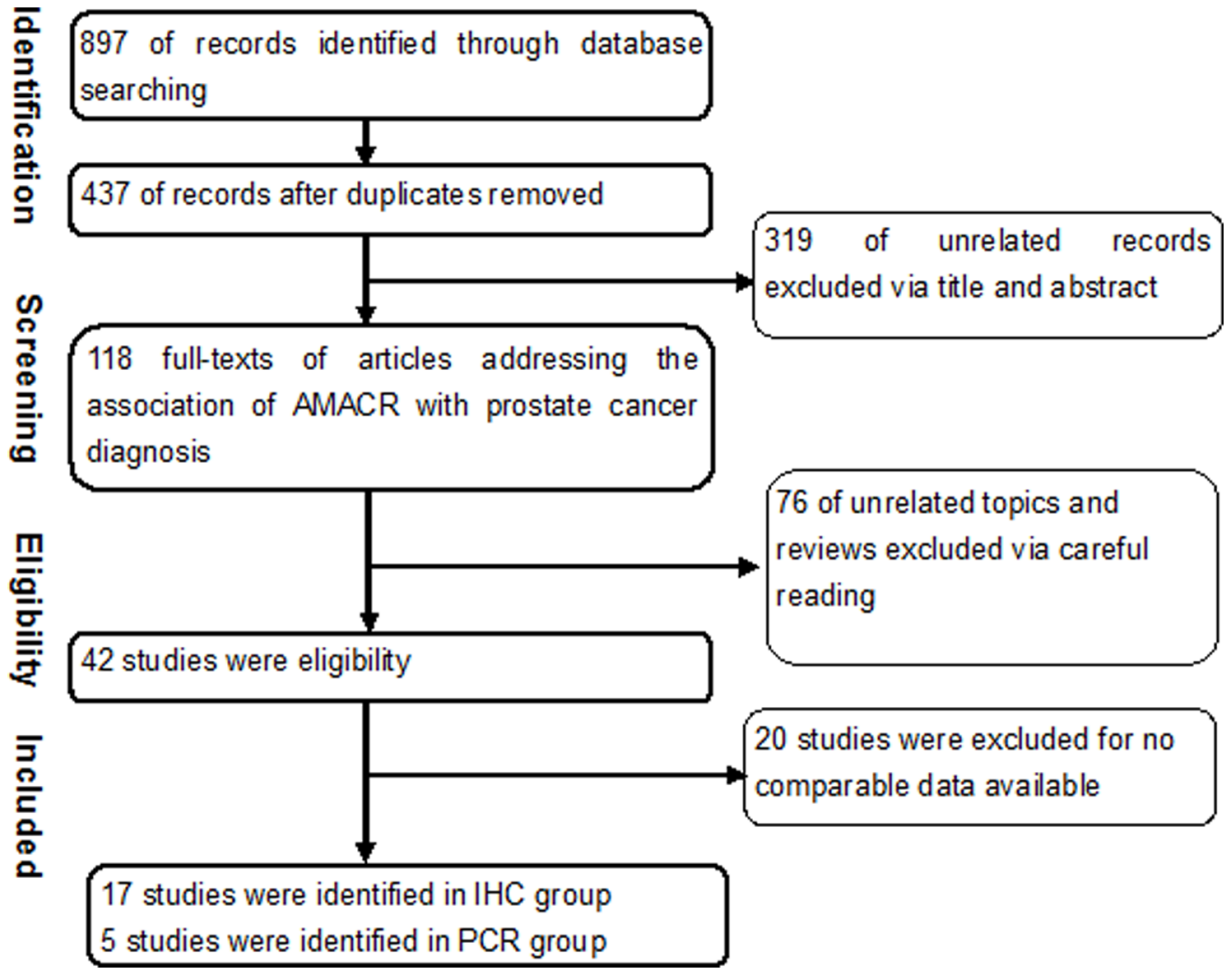
Flowchart of selecting process for meta-analysis.

The literature searches yielded a total 897 studies. After review of the abstracts, 118 studies were identified as potentially eligible for inclusion. After full review, 17 studies [14]–[30]using immunohistochemical method (IHC) and 5 studies [31]–[35] using Polymerase Chain Reaction (PCR) were deemed eligible and were included in the study. The list of studies excluded and reasons for exclusion are shown in Figure 1.

The included studies were published from 2004 to 2012. Five conducted in Asia, the others in western countries. Most of included studies chose benigh prostate hyperplasia. The details were listed in Table 1.

### Meta-analysis Results

**Figure 2:**
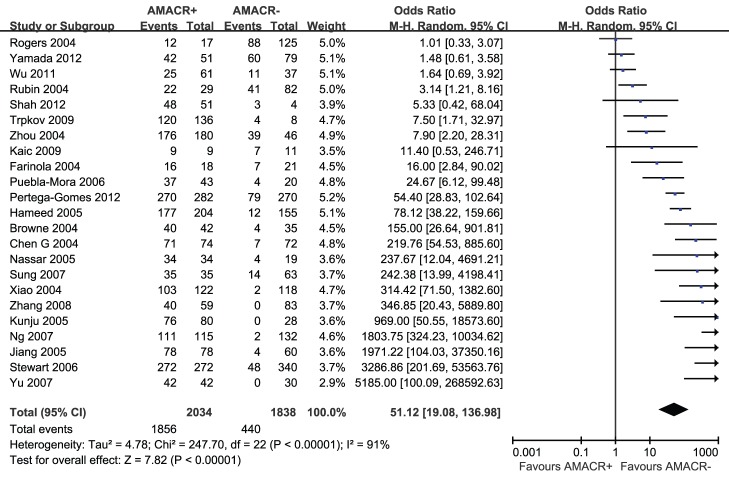
Forest plots for overall analysis of association of positive AMACR by immunohistochemistry with prostate cancer risk and under random-effects model. M-H = Mantel-Haenszel method; CI = confidence interval.

**Figure 3:**
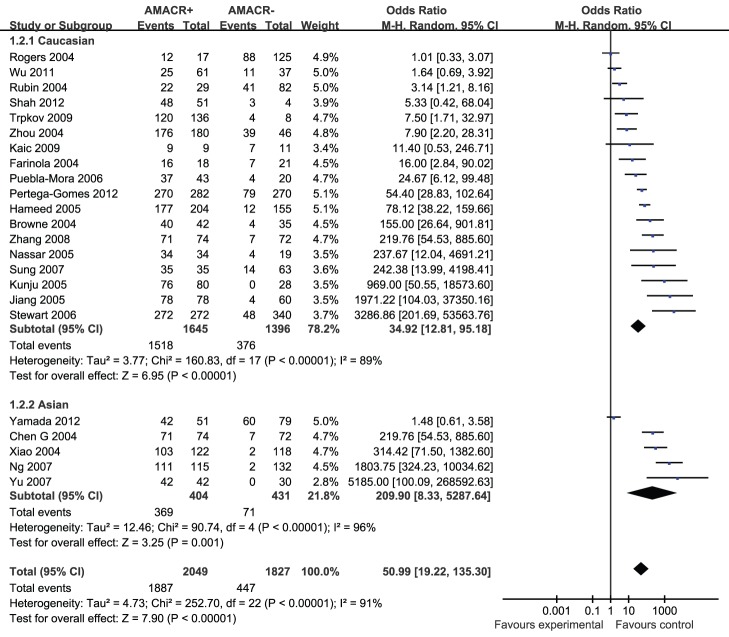
Forest plots for subgroup-analysis of association of positive AMACR by immunohistochemistry associated with prostate cancer risk in Caucasians and Asians. M-H = Mantel-Haenszel method; CI = confidence interval.

The pooled result revealed that positive AMACR by IHC was significantly associated with increased diagnosis of PCa (OR = 76.08; 95% CI, 25.53–226.68; P<0.00001) (Figure 2). Funnel plot asymmetry couldn’t be observed (Figure 3), which suggested no significant publication bias existing.

**Figure 4:**
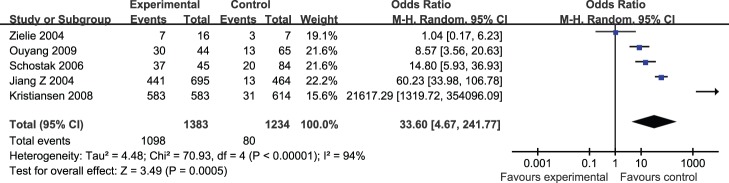
Forest plots for analysis of association of positive AMACR by RT-PCR with prostate cancer risk in; M-H = Mantel-Haenszel method; CI = confidence interval.

In consideration of the potential different expression of AMACR in different races, we yielded enthnicity-based subgroup-analyses (Figure 4). Subgroup-analysis showed that findings didn’t substantially change when only Caucasians (OR = 51.23; 95% CI, 19.41–135.24; P<0.00001), or Asians were included (OR = 209.90; 95% CI, 8.33–5287.64; P<0.00001). Both the results of subgroup-analyses showed that heterogeneity was usually a variation affecting the degree of risk rather than direction of effect.

**Figure 5:**
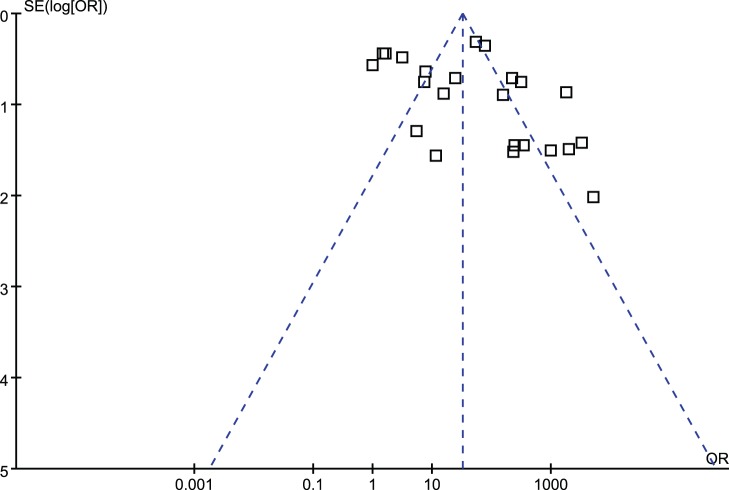
Funnel plots illustrating meta-analysis of overall analysis. SE = standard error; OR = odds ratio.

We next explored the positive AMACR by PCR in relation to PCa risk. Pooled results suggested that positive AMACR was associated with PCa (OR = 33.60; 95% CI, 4.67–241.77; P<0.00001) (Figure 5). There was also no significant publication bias observed.

## Discussion

In this study, we explored the association between the AMACR and PCa risk in 22 studies from various geographic regions including European and Asia. AMACR expression by IHC was significantly associated with increased diagnosis of PCa (OR = 76.08; 95% CI, 25.53–226.68; *P*<0.00001). The overall-analysis provided strong replication of the initial findings, confirming the AMACR for PCa.

AMACR is a well-characterized enzyme that plays a key role in peroxisomal b-oxidation of dietary branched fatty acids and C27-bile acid intermediates. It catalyzes the conversion of (R)-a-methyl-branched-chain fatty acyl-CoA esters to their (S)-stereoisomers. AMACR was identified as being overexpressed in prostate carcinoma cells when compared with benign or normal prostate epithelial cells [6]. The function of AMACR in prostate cancer has not been clarified yet. Several investigators have examined the mechanistic relationships between AMACR expression and hormone status. It has been reported that AMACR expression in hormone-sensitive cell lines and found its expression remained unchanged after exposure to antiandrogen drugs, suggesting that AMACR expression may not be directly regulated by the androgen pathway [36]. a-methylacyl-CoA racemase could not affect the stabilization of androgen receptor or modulate the expression of the androgen receptor–targeted gene, it indicating that the expression of AMACR is independent of androgen receptor–mediated signaling [37]. But Suzue et al [38] analyzed patients who had received hormonal therapy and found that those with localized prostate carcinoma had significantly diminished levels of AMACR expression. However, the exact mechanism by which hormonal therapy influences the expression level of AMACR remains elusive. Further studies are needed to further explore the mechanisms.

Strengths of this study include its large sample size. Because of this, the geographic regions were distinguished in subgroup-analyses. However, our results are based on unadjusted estimates, some un-provided parameters known to be associated with prostate carcinogenesis, such as inherent nature, might substantially confound the presented results.

## Conclusion

Meta-analysis of the comprehensive literature revealed that the AMACR expression was strongly associated with PCa risk in man from various regions. There was no varying between Caucasian and Asian man.

## Supporting Information

Checklist S1 **PRISMA checklist.**
(DOC)
