# Correction: A-Methylacyl-CoA Racemase (AMACR) and Prostate-Cancer Risk: A Meta-Analysis of 4,385 Participants

**DOI:** 10.1371/annotation/677fdf34-651e-4dc8-a0be-d0d633237a85

**Published:** 2013-12-17

**Authors:** Ning Jiang, Shimiao Zhu, Jing Chen, Yuanjie Niu, Liqun Zhou

In the second and third sentences of the Results subsection of the Abstract, the sentence should read: AMACR by IHC was significantly associated with increased diagnosis of PCa (OR=51.12; 95% CI, 19.08–136.98; P<0.00001). Subgroup-analysis showed that findings didn’t substantially change when only Caucasians or Asians (OR=34.92; 95% CI, 12.81–95.18; P<0.00001) were considered.

In the first sentence of the Meta-analysis Results subsection of the Evidence Synthesis, the sentence should read: he pooled result revealed that positive AMACR by IHC was significantly associated with increased diagnosis of PCa (OR=51.12; 95% CI, 19.08–136.98; P<0.00001) and Subgroup-analysis showed that findings didn’t substantially change when only Caucasians (OR=34.92; 95% CI, 12.81–95.18; P<0.00001).

In the second sentence of the first paragraph of the Discussion, the sentence should read: AMACR expression by IHC was significantly associated with increased diagnosis of PCa (OR=51.12; 95% CI, 19.08–136.98; P<0.00001). The overall analysis provided strong replication of the initial findings, confirming the AMACR for PCa. 

