# Supplementary material for: A-Methylacyl-CoA Racemase (AMACR) and Prostate-Cancer Risk: A Meta-Analysis of 4,385 Participants
Source: PLoS One. 2013 Oct 9;8(10):e74386. doi: 10.1371/journal.pone.0074386 (PMC3794046; doi:10.1371/journal.pone.0074386)
Supplement: Checklist S1 — PRISMA checklist. (DOC) [file pone.0074386.s001.doc]

| **Section/topic** | **#** | **Checklist item** | **Reported on page #** |
| --- | --- | --- | --- |
| **TITLE** | | |  |
| Title | 1 | **a-Methylacyl-CoA Racemase** **(AMACR) and Prostate-Cancer Risk : A Meta-analysis of 4,385 participants** | Title page |
| **ABSTRACT** | | |  |
| Structured summary | 2 | **Abstract**  **Introduction:** Alpha-methylacyl-CoA racemase (AMACR) is a mitochondrial and peroxisomal enzyme that is overexpressed in prostate cancer. The aim of this study was to conﬁrm and expand the findings that the PCa risk increased in men associated with AMACR expression across various geographic regions.  **Methods:** A systematic search of databases was carried out and other relevant articles were also identified. Then the meta-analyses were conducted according to the standard guidelines.  **Results:** A total of 22 studies with 4,385 participants were included on the basis of inclusion criterias. AMACR by IHC was signiﬁcantly associated with increased diagnosis of PCa (OR=76.08; 95% CI, 25.53-226.68; *P*<0.00001). Subgroup-analysis showed that ﬁndings didn’t substantially change when only Caucasians or Asians (OR=51.23; 95% CI, 19.41-135.24; *P*<0.00001) were considered. Expression of AMACR by PCR in relation to PCa risk suggested that AMACR was associated with PCa (OR=33.60; 95% CI, 4.67-241.77; *P*<0.00001). There was also no significant publication bias observed.  **Conclusion:** Our findings provide further evidences that the expression of AMACR contribute to PCa risk. AMACR protein overexpression was found in prostate cancers, low expression in any of the normal tissues or in benign prostatic tissue. AMACR is potentially an important prostate tumor marker. | Abstract |
| **INTRODUCTION** | | |  |
| Rationale | 3 | Numerous groups have now published their experience with the AMACR test in the diagnosis of gastric cancer, and varying results raise concerns about the AMACR as a biomarker in prostate cancer. | Introduction |
| Objectives | 4 | So own aim is to explore the potential value of AMACR in the diagnosis of prostate cancer by this meta-analysis, which, to our knowledge, has not previously been performed. | Introduction |
| **METHODS** | | |  |
| Protocol and registration | 5 | There is no review protocol existed. | None |
| Eligibility criteria | 6 | Diagnosis of prostate cancer glands can sometimes present a diagnostic challenge for pathologists, since prostate carcinoma can mimic benign prostate glands. and the architectural or cytologic clues for the diagnosis of carcinoma may not always be seen in small foci of suspicious glands. Also, Tissue diagnosis of prostate cancer can be difficult in needle biopsies or in a small focus of cancer of radical prostatectomies, presenting one of the major challenges in surgical pathology. underdiagnosis of a small focus of prostatic adenocarcinoma might delay early treatment and cause severe adverse consequences for patients. Therefore, a PCa specific marker could be be of great importance and usefulness to adjunct to facilitate critical diagnostic decisions with high sensitivity and specificity. | Materials and Methods |
| Information sources | 7 | Medline, Web of Science and the Cochrane Library | Materials and Methods |
| Search | 8 | The key words employed for literature retrieval are “AMACR” or “Alpha-methylacyl-CoA racemase” and “gastric” or “prostate” and “cancer” or “carcinoma” or “tumor” or “neoplasm” or “cancer” or “adenocarcinoma”. | Materials and Methods |
| Study selection | 9 | All publications identified by our search strategy were independently assessed by two reviewers. Disagreement about study selection was resolved by discussion and consensus. | Fig.1 |
| Data collection process | 10 | Two reviewers independently extracted data in every study to obtain information. | Materials and Methods |
| Data items | 11 | study design, age, gender, race, first author, year of publication, country of publication. | Materials and Methods |
| Risk of bias in individual studies | 12 | The funnel plot was addressed to reveal the potential publication bias. | Materials and Methods |
| Summary measures | 13 | The diagnostic meta-analysis was performed using a bivariate meta-analysis model to summarize the sensitivity, speciﬁcity, positive likelihood ratio (PLR), negative likelihood ratio (NLR), diagnostic odds ratio (DOR) in different races. | Materials and Methods |
| Synthesis of results | 14 | All analyses were conducted using Review Manage, version 5.2 (The Cochrane Collaboration, Oxford, U.K.) | Materials and Methods |

Page 1 of 2

| **Section/topic** | **#** | **Checklist item** | **Reported on page #** |
| --- | --- | --- | --- |
| Risk of bias across studies | 15 | publication bias, selective reporting within studies | Materials and Methods |
| Additional analyses | 16 | Sensitivity and subgroup analyses. | Materials and Methods |
| **RESULTS** | | |  |
| Study selection | 17 | In this study, we explored the association between the AMACR and PCa risk in 22 studies from various geographic regions including European and Asia. | Results |
| Study characteristics | 18 | age, gender, race, first author, year of publication, country of publication | Results, table 1 |
| Risk of bias within studies | 19 | The funnel plot was addressed to reveal the potential publication bias. The pooled result revealed that positive AMACR by IHC was signiﬁcantly associated with increased diagnosis of PCa (OR=76.08; 95% CI, 25.53-226.68; P<0.00001) (Figure 2). Funnel plot asymmetry couldn’t be observed (Figure 3), which suggested no significant publication bias existing. | none |
| Results of individual studies | 20 | In consideration of the potential different expression of AMACR in different races, we yielded enthnicity-based subgroup-analyses (Figure4). Subgroup-analysis showed that ﬁndings didn’t substantially change when only Caucasians (OR=51.23; 95% CI, 19.41-135.24; P<0.00001), or Asians were included (OR=209.90; 95% CI, 8.33-5287.64; P<0.00001). Both the results of subgroup-analyses showed that heterogeneity was usually a variation affecting the degree of risk rather than direction of effect. | Results |
| Synthesis of results | 21 | the Odds Ratio: OR=76.08; 95% CI, 25.53-226.68; P<0.00001. Caucasians (OR=51.23; 95% CI, 19.41-135.24; P<0.00001), Asians(OR=209.90; 95% CI, 8.33-5287.64; P<0.00001). AMACR by PCR(OR=33.60; 95% CI, 4.67-241.77; P<0.00001) | Results |
| Risk of bias across studies | 22 | NA | none |
| Additional analysis | 23 | AMACR by PCR(OR=33.60; 95% CI, 4.67-241.77; P<0.00001) | Results |
| **DISCUSSION** | | |  |
| Summary of evidence | 24 | he pooled result revealed that positive AMACR by IHC was signiﬁcantly associated with increased diagnosis of PCa (OR=76.08; 95% CI, 25.53-226.68; P<0.00001). Funnel plot asymmetry couldn’t be observed , which suggested no significant publication bias existing. | Discussion |
| Limitations | 25 | Strengths of this study include its large sample size. Because of this, the geographic regions were distinguished in subgroup-analyses. However, our results are based on unadjusted estimates, some un-provided parameters known to be associated with prostate carcinogenesis, such as inherent nature, might substantially confound the presented results. | Discussion |
| Conclusions | 26 | Meta-analysis of the comprehensive literature revealed that the AMACR expression was strongly associated with PCa risk in man from various regions. There was no varying between Caucasian and Asia man. | Discussion |
| **FUNDING** | | |  |
| Funding | 27 | Grant sponsor: Science Foundation of Tianjin (NO:11JCZDJC19700), 2010KZ95 and Grant numbers: 09ZCZDSF04300; the National Natural Science Foundation of China Grant numbers: 2012CB518304,2012DFG32220. | online submission system |

*From:*  Moher D, Liberati A, Tetzlaff J, Altman DG, The PRISMA Group (2009). Preferred Reporting Items for Systematic Reviews and Meta-Analyses: The PRISMA Statement. PLoS Med 6(6): e1000097. doi:10.1371/journal.pmed1000097

For more information, visit: **www.prisma-statement.org**.

Page 2 of 2
